# Lower Serum *n*-3 Fatty Acid Level in Older Adults with Sarcopenia

**DOI:** 10.3390/nu12102959

**Published:** 2020-09-27

**Authors:** Il-Young Jang, Hee-Won Jung, Jin Hoon Park, Jeoung Hee Kim, Seungjoo Lee, Eunju Lee, Jin Young Lee, So Jeong Park, Da Ae Kim, Su Jung Kim, Hyun Ju Yoo, Beom-Jun Kim

**Affiliations:** 1 Division of Geriatrics, Department of Internal Medicine, Asan Medical Center, University of Ulsan College of Medicine, Seoul 05505, Korea; onezero2@gmail.com (I.-Y.J.); dr.ecsta@gmail.com (H.-W.J.); eunjulee@amc.seoul.kr (E.L.); 2 Department of Neurological Surgery, Asan Medical Center, University of Ulsan College of Medicine, Seoul 05505, Korea; spinejhpark@naver.com (J.H.P.); jeounghee@amc.seoul.kr (J.H.K.); changhill@gmail.com (S.L.); 3 Asan Institute for Life Sciences, Asan Medical Center, University of Ulsan College of Medicine, Seoul 05505, Korea; takewlsdud@gmail.com (J.Y.L.); sjpark99@hanmail.net (S.J.P.); sizzzeri@gmail.com (D.A.K.); 4 Department of Convergence Medicine, Asan Institute for Life Sciences, Asan Medical Center, University of Ulsan College of Medicine, Seoul 05505, Korea; su-jungea@hanmail.net; 5 Division of Endocrinology and Metabolism, Department of Internal Medicine, Asan Medical Center, University of Ulsan College of Medicine, Seoul 05505, Korea

**Keywords:** *n*-3 fatty acid, sarcopenia, anti-inflammation, aging, biomarker

## Abstract

The *n*-3 fatty acid (FA) has evoked considerable interest as a modifiable factor for maintenance of muscle health owing to its anti-inflammatory properties. To clarify this possibility, we investigated circulating *n*-3 FA level, a reliable biomarker of FA status in the body, in relation to sarcopenia in a cohort of Asian older adults. Blood samples were collected from 125 participants who underwent comprehensive assessment of muscle mass and function. Serum FA level was measured by gas chromatography/mass spectrometry. Sarcopenia was diagnosed using the cut-off points specified for the Asian population. After adjusting for sex, age, and body mass index, subjects with sarcopenia and those with low muscle strength had 36.5% and 32.4% lower serum *n*-3 levels (*P* = 0.040 and 0.030), respectively, than controls. The odds ratios per standard deviation increment in serum *n*-3 level for sarcopenia and low muscle strength were 0.29 and 0.40 (*P* = 0.015 and 0.028), respectively. A higher serum *n*-3 level was significantly associated with greater muscle strength (*P* = 0.038). These findings suggest a possible protective effect of *n*-3 FA on human muscle homeostasis. Further well-designed large-scale longitudinal studies are necessary to understand the definite role of circulating *n*-3 FA level in sarcopenia risk assessment.

## 1. Introduction

The term sarcopenia refers to the age-related loss of skeletal muscle mass and strength resulting from the imbalance between protein breakdown and synthesis [1,2]. Sarcopenia has received increasing attention in geriatrics, especially during the last decade. Studies have shown a consistent link of sarcopenia with higher mortality as well as adverse health outcomes, such as comorbidities, disability, loss of independence, and poor quality of life [3,4,5,6]. Owing to the progressive population aging, sarcopenia is likely to become a much more serious public health concern in the future. Sarcopenia has long been believed to be an inevitable consequence of aging; however, owing to the recent advances in knowledge about muscle metabolism, it is now regarded as a disease that is amenable to intervention [7]. Therefore, continuous efforts to slow or reverse sarcopenia are critical to achieve the goal of increasing the healthy life span of older adults.

Physical exercises and appropriate nutritional support are generally recommended as non-pharmacological approaches for the prevention and treatment of sarcopenia [8]. Traditional nutritional interventions have largely focused on increasing the protein intake [9,10]; however, accumulating evidence now suggests the potential role of *n*-3 polyunsaturated fatty acid (PUFA) in muscle health [11]. The *n*-3 fatty acid (FA) is an essential nutrient that cannot be synthesized in mammals; therefore, this should be obtained from diets and nutritional supplements [12]. Overall, *n*-3 FA exerts protective effects against chronic metabolic diseases via its anti-inflammatory property [13,14]. Considering that age-associated low-grade inflammation, also referred to as “inflamm–aging”, plays a critical role in the pathogenesis of sarcopenia [15,16], the *n*-3 FA may potentially have a beneficial effect on muscle metabolism in humans. To test this hypothesis, we investigated the circulating level of *n*-3 FA and assessed its relation with sarcopenia and related parameters in a cohort of Asian older adults.

## 2. Materials and Methods

### 2.1. Study Participants

The study population consisted of South Koreans who visited the Division of Geriatrics, Department of Internal Medicine, Asan Medical Center (Seoul, Korea), to undergo comprehensive geriatric assessment between April 2019 and April 2020. Participants whose life expectancy was less than 1 year due to malignancy and those with symptomatic heart failure or end-stage renal disease were excluded. After exclusion of ineligible participants, blood samples were collected from 125 eligible participants. The study was approved by the center’s Institutional Review Board (no. 2020–0259). Written informed consent was obtained from all participants prior to their enrolment.

### 2.2. Sarcopenia Assessment

Information pertaining to demographic characteristics and medical and surgical history was collected through detailed interviews and medical record review conducted by experienced nurses. Body composition including muscle mass (whole body lean body mass minus bone mineral content) was evaluated using a bioelectrical impedance analyzer (InBody S10; InBody, Seoul, Korea) with measuring frequencies of 1, 5, 50, 250, 500, and 1000 kHz [17]. Appendicular skeletal muscle mass (ASM) was calculated as the sum of the muscle mass of both arms and legs. The skeletal muscle mass index (SMI) was defined by adjusting the ASM relative to the height squared to ensure an objective comparison of muscle mass between participants [18]. Handgrip strength of the dominant side was measured using a Jamar hydraulic hand dynamometer (Patterson Medical, Warrenville, IL, USA) [19]. Participants were instructed to sit comfortably, bend their elbow at 90 degrees, and grip the dynamometer as firmly as possible. The maximum value was adjusted after all tests were conducted twice at 1-min intervals or longer. In addition, all subjects underwent the short physical performance battery (SPPB) using electronic SPPB toolkit (eSPPB) developed by Dyphi Inc. (Daejoen, Korea); it consisted of repeated chair stands, standing balance, and walking speed [20]. In the 5-time chair stand test, the time required by participants to complete 5 sit-to-stand maneuvers as quickly as possible was recorded. In the standing balance test, which included a side-by-side stance, semi-tandem stance, and a tandem stance, the subjects were instructed to stand for up to 10 s. For walking speed, a 4-m usual gait speed with a separate 1-m acceleration distance that was not included in the speed calculation was acquired [21,22]. The SPPB total score was calculated by combining these 3 components (range: 0 (worst) to 12 (best)).

Sarcopenia was diagnosed using the 2019 Consensus Guidelines of the Asian Working Group for sarcopenia [1]. Briefly, older patients with low muscle mass (SMI < 7.0 kg/m^2^ for men and <5.7 kg/m^2^ for women) and low muscle strength (handgrip strength < 28 kg for men, <18 kg for women) and/or low physical performance (gait speed < 1.0 m/s, 5-time chair stand test ≥ 12 s, or SPPB score ≤ 9 points) were classified as having sarcopenia.

### 2.3. Measurement of Serum Levels of n-3 and n-6 FAs

Blood samples were collected from the antecubital vein in the morning after an overnight fast of at least 8 h. After centrifugation of samples at 3000 rpm for 5 min at 4 °C, the supernatant was carefully collected to exclude the cellular component. All samples that showed hemolysis or clotting were discarded. The serum samples were stored at −80 °C prior to measuring the concentrations. To determine the concentrations of FAs, 50 μL of serum was combined and mixed well with 200 μL of a chloroform/methanol mixture (1:2, *v/v*) and 50 μL of an internal standard solution (0.1 mg/mL myristic acid-d_14_). After centrifugation at 2000× *g* for 15 min, the lower organic phase was collected and dried. The dried sample was hydrolyzed with 200 μL of 0.5 M KOH in MeOH at 80 °C for 30 min, cooled to room temperature, and then reacted with 200 μL of 12% (*w/w*) BCl_3_-MeOH (Sigma-Aldrich, St. Louis, MO, USA) at 60 °C for 30 min. Subsequently, 100 μL of H_2_O and 100 μL of hexane were added sequentially and mixed vigorously. After 5 min of rest, the upper lipid phase was collected and added to 20–30 mg of anhydrous sodium sulfate to remove traces of water, and the supernatant was subjected to gas chromatography/mass spectrometry analysis. FA methyl esters (Sigma-Aldrich) without derivatization were used to generate calibration curves. The concentration of *n*-3 FA was calculated by summing the eicosapentaenoic acid (C20:5 *n*-3) and docosahexaenoic acid (C22:6 *n*-3) contents, while the concentration of *n*-6 FA was calculated by summing the linoleic acid (C18:2 *n*-6), arachidonic acid (C20:4 *n*-6), and eicosatrienoic acid (C20:3 *n*-6) contents. Although C18:3 *n*-6 and C22:5 *n*-6 were also observed in human serum, their amounts were negligible compared to the total amount of C18:2 *n*-6, C20:3 *n*-6, and C20:4 *n*-6 (Figure S1). The inter-assay coefficients of variation (CVs) for C18:2 *n*-6, C20:3 *n*-6, C20:4 *n*-6, C20:5 *n*-3, and C22:6 *n*-3 were less than 5% with few exceptions; all values were within the acceptability criterion of 15% for reproducible assays (Table S1).

### 2.4. Statistical Analysis

All clinical data are presented as mean ± standard deviation (SD) or as frequencies and percentages, unless specified otherwise. The baseline characteristics of subjects with and without sarcopenia were compared using Student’s *t*-test for the continuous variables and Chi-squared test for the categorical variables. The estimated mean values with 95% confidence intervals of serum levels of *n*-3 and *n*-6 FAs according to the status of sarcopenia and related parameters were generated and compared using the analysis of covariance (ANCOVA) after adjusting for sex, age, and body mass index (BMI). Logistic regression analysis was performed to generate the odds ratio (OR) for sarcopenia and poor muscle outcomes according to serum levels of *n*-3 and *n*-6 FAs. The association of serum levels of *n*-3 and *n*-6 FAs with sarcopenia parameters was assessed using linear regression analysis. All statistical analyses were performed using the Statistical Package for the Social Sciences (SPSS) version 18.0 (SPSS Inc., Chicago, IL, USA). *P* values < 0.05 were considered indicative of statistical significance.

## 3. Results

Table 1 lists the baseline characteristics of the study population. Out of the 125 subjects, 21 had sarcopenia while 104 did not have sarcopenia; the number of women in the two groups was 14 (66.7%) and 54 (51.9%), respectively. The mean age of subjects with and without sarcopenia was 71.9 ± 4.7 and 68.6 ± 6.5 years, respectively. Compared with the subjects without sarcopenia, those with sarcopenia had lower weight, height, BMI, ASM, SMI, grip strength, gait speed and SPPB score; in addition, subjects with sarcopenia required a longer time to complete five chair stands.

**Figure 1:**
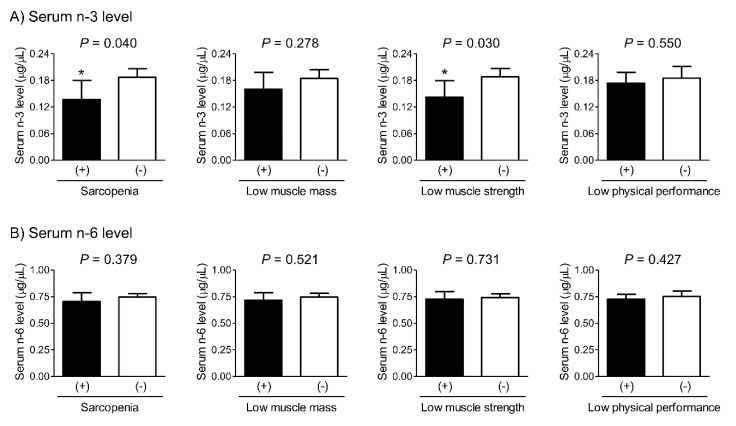
Differences in serum levels of *n*-3 (**A**) and *n*-6 (**B**) fatty acids according to the status of sarcopenia and related parameters after adjusting for sex, age, and BMI. * indicates a statistically significant difference from control. The estimated mean values with 95% confidence intervals were generated and compared using analysis of covariance. BMI, body mass index.

Differences in serum levels of *n*-3 and *n*-6 FAs according to the status of sarcopenia and related parameters were assessed using ANCOVA (Figure 1). After adjusting for sex, age, and BMI, subjects with sarcopenia and low muscle strength had 36.5% and 32.4% lower serum *n*-3 levels, respectively, than those without these conditions. However, no significant between-group difference was observed with respect to serum *n*-6 levels. 

The risk of sarcopenia and poor muscle outcomes was determined by logistic regression analysis (Table 2). After adjusting for potential confounders, the ORs for sarcopenia and low muscle strength per SD increment in serum *n*-3 level were 0.29 and 0.40, respectively. However, the ORs for sarcopenia and related parameters in terms of serum *n*-6 level were not statistically significant.

Linear regression analysis was performed to examine the relationship of serum levels of *n*-3 and *n*-6 FAs with specific muscle parameters related to sarcopenia (Table 3). After adjusting for sex, age, and BMI, serum *n*-3 FA concentration showed a positive association with grip strength. However, serum *n*-6 level showed no correlation with any of the muscle parameters.

Although we used the absolute concentration of serum *n*-3 and *n*-6 FAs in the analyses above, the relative value after considering total FAs has been also widely used for research. Therefore, serum *n*-3 and *n*-6 FAs were expressed as the percentage of serum total FAs, and then compared according to the status of sarcopenia and related parameters. As shown in Figure S2, after adjusting for sex, age, and BMI, subjects with sarcopenia and low muscle strength had significantly lower serum *n*-3 percentage than controls. However, no significant between-group difference was observed with respect to serum *n*-6 percentage. Collectively, regardless of whether it was the absolute concentration or the relative value, we could observe that serum *n*-3 FA was markedly lower in subjects with sarcopenia and low muscle strength. 

## 4. Discussion

Most previous human studies have investigated the association of *n*-3 and *n*-6 FAs only with specific muscle parameters, such as muscle mass or quality [23,24,25,26,27]. However, in the present study, we focused on sarcopenia, which combines various muscle phenotypes, using the well-accepted diagnosis algorithm in a cohort of older adults. In our study, subjects with sarcopenia had markedly lower serum *n*-3 level than those without sarcopenia after adjusting for sex, age, and BMI. Furthermore, the risk of sarcopenia was decreased by 71% per SD increment in serum *n*-3 level. To the best of our knowledge, this study provides the first clinical evidence of the potential relation between the circulating levels of certain PUFAs (especially *n*-3) and sarcopenia, and supports the hypothesis that *n*-3 FA may act as a modifiable factor for the maintenance of muscle health in Asian older adults.

Based on the experiments that showed the beneficial effects of *n*-3 FA on muscle metabolism [28,29], several epidemiological studies have investigated the link between *n*-3 FA intake and muscle parameters [24,25,26,30,31,32]. However, the results were largely conflicting probably due to the methodological limitations. For example, the use of self-reported recall method (using food frequency questionnaire) is liable to introduce an element of reporting bias which limits the accuracy of results. In addition, FA supplements (such as fish oil) are highly heterogenous and contain other bioactive components [33] that may also affect muscle metabolism; this may have also contributed to the inconsistent results. Therefore, the use of objective indicators of dietary intake or supplementation is a key imperative in clinical research related to PUFAs. Currently, PUFAs are frequently analyzed in adipose tissue and blood fractions including red blood cells (RBCs) or serum. Although we used human serum because of possible allocation for other analytical measurements and convenience and cost of sample preparation, RBC FAs are known to have the longest half-life and reflect the long-term dietary intake of *n*-3 FA [34]. Therefore, the association of serum *n*-3 FA with sarcopenia observed in our study needs to be validated using samples from RBCs.

The precise mechanism of the beneficial effect of *n*-3 FA on muscle metabolism is yet to be elucidated; however, there are several plausible mechanisms. First, the anti-inflammatory property of *n*-3 FA has been the most frequently suggested mechanism [35,36]. Chronic inflammation resulting from dysregulation of the immune system and age-related increase in pro-inflammatory cytokines (such as IL-6 and TNF-α) is known to contribute to muscle wasting [37,38]. Therefore, alleviation of inflammation by *n*-3 FAs is a fairly plausible mechanism of its beneficial effects against sarcopenia. Second, *n*-3 FA may directly increase the rate of muscle protein synthesis through the activation of mTOR signals [39]. The mTOR pathway is a key anabolic factor that mediates skeletal muscle generation. Interestingly, some studies have shown that stimulation of mTOR and its downstream regulators by *n*-3 FA may inhibit age-related muscle loss [29,40]. Third, *n*-3 FA may contribute to the maintenance of muscle health via the activation of satellite cells, which are primarily responsible for muscle regeneration after damaging stimuli [39,41]. Fourth, *n*-3 FA may increase the muscle strength and functional capacity by improving neuromuscular junction conductivity and muscle contractile activity [42,43].

An interesting finding of this study was that serum *n*-6 FA level did not show a significant association with sarcopenia. The *n*-3 and *n*-6 FAs are distinguished by the location of the first double bond from the methyl (ω) end of the molecule; in addition, these cannot be endogenously converted to each other. Importantly, unlike the *n*-3 FA, it is less clear whether the *n*-6 FA is protective or harmful on chronic metabolic diseases [44,45,46]. Consistently, the role of *n*-6 FA on human muscle metabolism could not be inferred from our study.

Despite recognized pleiotropic effects of *n*-3 FA on human health, the amount of *n*-3 FA that would be necessary for the purpose of preventing or treating specific diseases might be too high to be consumed only by diets or supplements. Interestingly, recent animal studies demonstrated that G protein-coupled receptor 120 (GPR120; also known as free FA receptor 4) can mediate the action of *n*-3 FA in various tissues and organs including musculoskeletal systems [47,48]. Therefore, the development of high-affinity small molecules targeting GPR120 is expected to contribute to the maintenance of muscle health during aging.

The major strength of our study is that we adopted the Asian-specific cut-off point for the diagnosis of sarcopenia [1], because the muscle parameters may vary depending on ethnicity, body size, lifestyle, and cultural background. We also included all the recommended diagnostic criteria for sarcopenia according to the guidelines, such as muscle mass, handgrip strength, gait speed, 5-time chair stand test, and SPPB [1]; this helped increase the reliability of our results. However, despite these strengths, several limitations should be considered while interpreting our data. First, the cross-sectional study design does not permit causal inferences with respect to the relationship between serum *n*-3 FA level and sarcopenia. Although we assume that low *n*-3 FA may increase the chance of sarcopenia based on the previous experiments showing the protective roles of *n*-3 FA on muscle metabolism [28,29], there is the possibility that our results could have been generated due to a reverse causal relationship, which implies that low serum *n*-3 FA levels may be attributable to altered PUFA metabolism in subjects with sarcopenia. Second, the study population consisted of individuals who visited a hospital; thus, these may not be representative of the general population, possibly resulting in selection bias. Third, supplementary and dietary FA intakes could not be evaluated in this study. Although these factors would be reflected in serum FA levels and thus may not be critical confounding factors, the lack of this information in this cohort should be regarded as one of the limitations. Lastly, we cannot exclude the possibility that our findings could have been biased by uncontrolled factors that affect serum FA level and/or muscle metabolism, such as 25-hydroxyvitamin D.

In conclusion, we observed that lower serum *n*-3 level was significantly associated with a higher risk of sarcopenia in older adults after controlling for potential confounders. These findings suggest a possible protective effect of *n*-3 FA on human muscle homeostasis. Further well-designed large-scale longitudinal studies are necessary to understand the definite role of circulating *n*-3 FA level in sarcopenia risk assessment.

## Figures and Tables

**Table 1:** Baseline characteristics of the study population according to sarcopenia status.

Variables	Sarcopenia (*n* = 21)	No Sarcopenia (*n* = 104)	*P*
Sex, n (%)			0.216
Female	14 (66.7)	54 (51.9)	
Male	7 (33.3)	50 (48.1)	
Age, years	**71.9 ± 4.7**	**68.6 ± 6.5**	**0.034**
Weight, kg	**58.6 ± 9.6**	**68.0 ± 10.1**	**<0.001**
Height, cm	**156.0 ± 5.7**	**160.0 ± 9.3**	**0.011**
Body mass index, kg/m^2^	**24.0 ± 3.3**	**26.5 ± 3.1**	**0.001**
ASM, kg	**13.4 ± 2.6**	**18.3 ± 4.7**	**<0.001**
SMI, kg/m^2^	**5.48 ± 0.78**	**7.03 ± 1.06**	**<0.001**
Grip strength, kg	**21.9 ± 6.2**	**28.9 ± 9.3**	**<0.001**
Gait speed, m/s	**0.87 ± 0.37**	**1.10 ± 0.37**	**0.012**
Chair stand, s	**15.9 ± 11.4**	**10.4 ± 6.4**	**0.047**
SPPB score (range, 0–12)	**9.2 ± 2.6**	**10.8 ± 1.7**	**0.001**

Data presented as mean ± standard deviation, unless otherwise specified. **Bold** indicates statistically significant values. Differences between the two groups were assessed using Student’s *t*-test for continuous variables and Chi-squared test for categorical variables. ASM, appendicular skeletal muscle mass; SMI, skeletal muscle mass index; SPPB, short physical performance battery.

**Table 2:** Logistic regression analyses to determine the odds ratios for sarcopenia and related parameters according to serum levels of *n*-3 and *n*-6 fatty acids.

	OR (95% CIs) per SD Increment in Serum *n*-3 Level	*P*	OR (95% CIs) per SD Increment in Serum *n*-6 Level	*P*
Sarcopenia	**0.29 (0.11–0.79)**	**0.015**	0.84 (0.49–1.45)	0.537
Low muscle mass	0.68 (0.35–1.33)	0.258	0.90 (0.55–1.45)	0.649
Low muscle strength	**0.40 (0.18–0.91)**	**0.028**	0.93 (0.58–1.49)	0.749
Low physical performance	0.89 (0.61–1.30)	0.545	0.85 (0.57–1.26)	0.417

**Bold** values are statistically significant after adjusting for sex, age, and body mass index. OR, odds ratio; SD, standard deviation.

**Table 3:** Linear regression analyses to determine the association of serum levels of *n*-3 and *n*-6 fatty acids with sarcopenia parameters.

	Serum *n*-3 Level				Serum *n*-6 Level			
	β	SE	*β*	*P*	β	SE	*β*	*P*
SMI	1.153	0.761	0.096	0.132	0.068	0.408	0.011	0.867
Grip strength	**13.130**	**6.261**	**0.138**	**0.038**	2.634	3.378	0.053	0.437
Gait speed	0.215	0.332	0.055	0.518	–0.001	0.177	–0.001	0.994
Chair stand	−6.814	6.973	–0.086	0.330	1.522	3.716	0.036	0.683
SPPB score	1.675	1.781	0.083	0.349	0.034	0.950	0.003	0.971

**Bold** values are statistically significant after adjusting for sex, age, and body mass index. SMI, skeletal muscle mass index; SPPB, short physical performance battery; β, unstandardized regression coefficient; SE, standard error; *β*, standardized regression coefficient.

## Supplementary Materials

The following are available online at https://www.mdpi.com/2072-6643/12/10/2959/s1, **Figure S1.** Total ion chromatogram of fatty acid methyl esters from human serum. **Table S1.** Accuracy and inter-assay coefficients of variation for each fatty acid. **Figure S2.** Differences in serum *n*-3/total fatty acid and serum *n*-6/total fatty acid ratio according to the status of sarcopenia and related parameters after adjusting for sex, age, and body mass index.
