# Supplementary material for: Lower Serum n-3 Fatty Acid Level in Older Adults with Sarcopenia
Source: Nutrients. 2020 Sep 27;12(10):2959. doi: 10.3390/nu12102959 (PMC7600475; doi:10.3390/nu12102959)
Supplement: Supplementary file 1 [file nutrients-12-02959-s001.pdf]

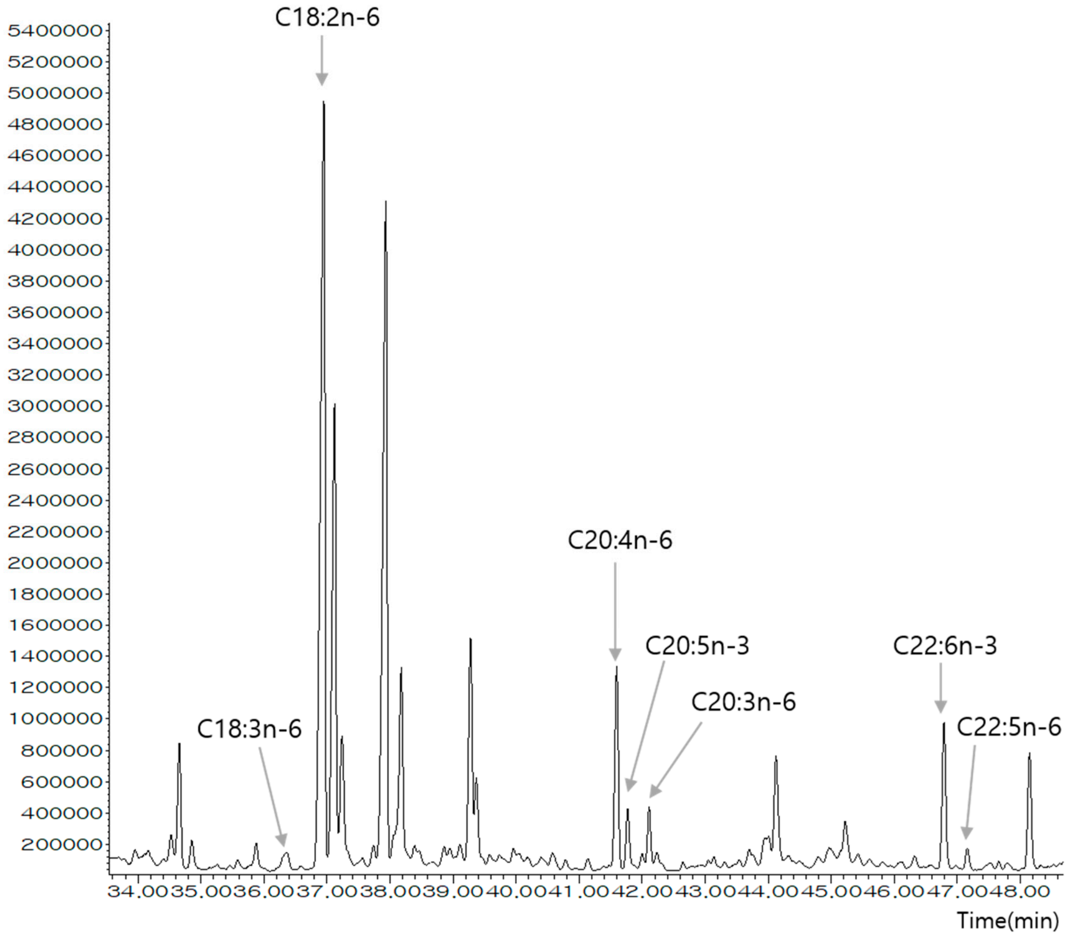

Figure S1. Total ion chromatogram of fatty acid methyl esters from human serum. Very small amount of C18:3 n-6 and C22:5 n-6 existed in human serum, comparing to the total amounts of C18:2 n-6, C20:4 n-6, and C20:3 n-6. The peaks of C18:3 n-6 were close to the lower range of calibration curve and often below the limit of quantitation. C22:5 n-6 were not contained in 37 FAME mix (Sigma-Aldrich), and calibration curve could not generated. The area ratio (the peak area of 22:5 n-6 divided by that of IS) could be obtained.

Table S1. Accuracy and inter-assay coefficients of variation for each fatty acid

Standard solution		C18:2 n-6		C20:3 n-6		C20:4 n-6		C20:5 n-3		C22:6 n-3	
		Accuracy (%)	CV	Accuracy (%)	CV	Accuracy (%)	CV	Accuracy (%)	CV	Accuracy (%)	CV
Concentration (mg/mL)	2.5	108.3	4.7	115.8	2.2	103.7	2.4	113.9	5.6	99.7	3.2
	9.0	100.6	3.8	96	11.2	101	4.4	108.1	13.4	101.5	5

Independent experiments were preformed over three days using standard solutions. The concentration of the standard solutions was selected within the respective calibration range (0.5–10 mg/mL). The raw signal of each fatty acid from sample solutions was higher than 1.0 mg/mL; if necessary, the sample solutions were diluted to fall within the calibration range of each fatty acid. CV, coefficient of variation.

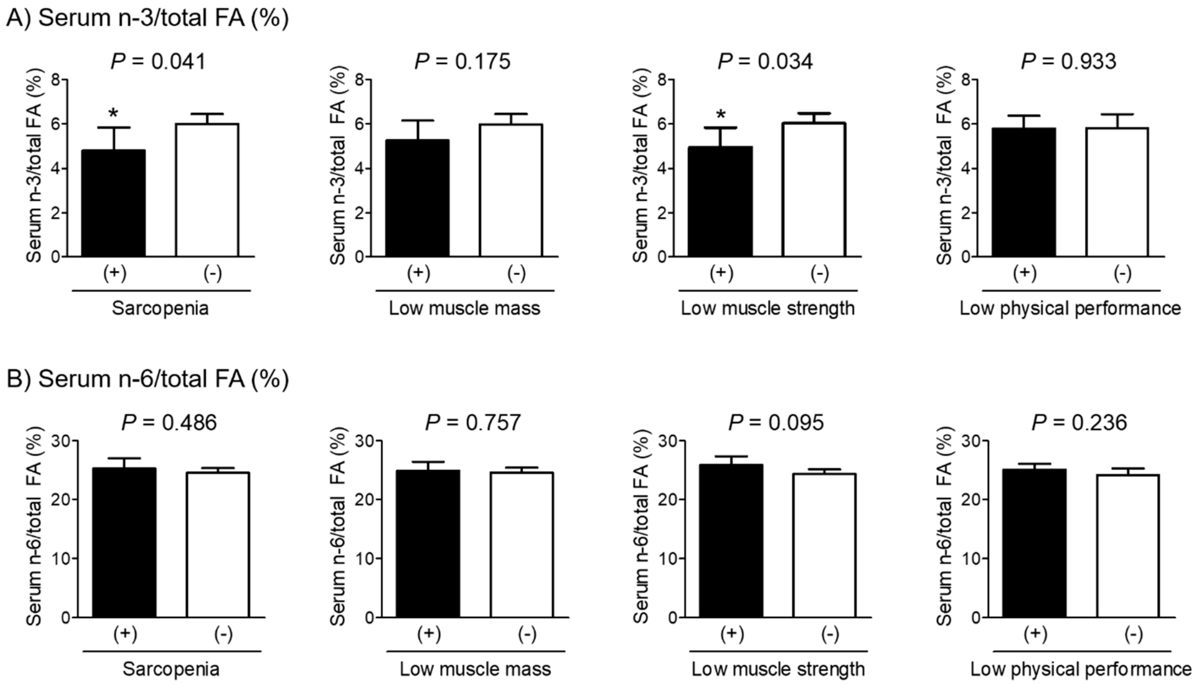

Figure S2. Differences in serum n-3/total FA (A) and n-6/total FA ratio (B) according to the status of sarcopenia and related parameters after adjusting for sex, age, and BMI. The estimated mean values with 95% confidence intervals were generated and compared using analysis of covariance. BMI, body mass index; FA, fatty acid.
